# Structure-Based Design of Novel MAO-B Inhibitors: A Review

**DOI:** 10.3390/molecules28124814

**Published:** 2023-06-16

**Authors:** Emilio Mateev, Maya Georgieva, Alexandrina Mateeva, Alexander Zlatkov, Shaban Ahmad, Khalid Raza, Vasco Azevedo, Debmalya Barh

**Affiliations:** 1 Department of Pharmaceutical Chemistry, Faculty of Pharmacy, Medical University-Sofia, 1000 Sofia, Bulgaria; e.mateev@pharmfac.mu-sofia.bg (E.M.); a.dineva@pharmfac.mu-sofia.bg (A.M.); azlatkov@pharmfac.mu-sofia.bg (A.Z.); 2 Department of Computer Science, Jamia Millia Islamia, New Delhi 110025, India; shaban184343@st.jmi.ac.in (S.A.); kraza@jmi.ac.in (K.R.); 3 Departamento de Genetica, Ecologia e Evolucao, Instituto de Ciências Biológicas, Universidade Federal de Minas Gerais, Belo Horizonte 31270-901, Brazil; vascoariston@gmail.com (V.A.); dr.barh@gmail.com (D.B.); 4 Institute of Integrative Omics and Applied Biotechnology (IIOAB), Nonakuri, Purba Medinipur 721172, India

**Keywords:** molecular docking, structure-based drug design, MAO-B inhibitors, chalcones, coumarins, chromones, xanthines, pyrazoles

## Abstract

With the significant growth of patients suffering from neurodegenerative diseases (NDs), novel classes of compounds targeting monoamine oxidase type B (MAO-B) are promptly emerging as distinguished structures for the treatment of the latter. As a promising function of computer-aided drug design (CADD), structure-based virtual screening (SBVS) is being heavily applied in processes of drug discovery and development. The utilization of molecular docking, as a helping tool for SBVS, is providing essential data about the poses and the occurring interactions between ligands and target molecules. The current work presents a brief discussion of the role of MAOs in the treatment of NDs, insight into the advantages and drawbacks of docking simulations and docking software, and a look into the active sites of MAO-A and MAO-B and their main characteristics. Thereafter, we report new chemical classes of MAO-B inhibitors and the essential fragments required for stable interactions focusing mainly on papers published in the last five years. The reviewed cases are separated into several chemically distinct groups. Moreover, a modest table for rapid revision of the revised works including the structures of the reported inhibitors together with the utilized docking software and the PDB codes of the crystal targets applied in each study is provided. Our work could be beneficial for further investigations in the search for novel, effective, and selective MAO-B inhibitors.

## 1. Introduction

Neurodegenerative disorders (NDs) are age-dependent diseases with high morbidity rates and complex pathophysiology. With the prediction that NDs could place second for the most prevalent cause of death [1], there is an urgent need for novel, effective and safe medicines. Two common NDs are Alzheimer’s and Parkinson’s disease, which are briefly reviewed here. Parkinson’s disease (PD) is mostly associated with motor disabilities caused by the depletion of dopamine within the substantia nigra (SN). The SN is a midbrain dopaminergic nucleus that plays a critical role in modulating motor movement and reward functions as part of the basal ganglia circuitry. PD could also affect the cognitive functions of the brain that may cause depression and sleep problems [2,3]. A recent review, published by Oertel and Schulz [4], expressed the importance of MAO-B (monoamine oxidases B) inhibitors as additive treatment in PD. Alzheimer’s disease (AD) is defined as a progressive and persistent illness that leads to personality changes and cognitive impairments [5]. A recent study by Schedin-Weiss et al. [6] showed elevated levels of MAO-B in astrocytes and pyramidal neurons in patients with AD. Hence, compounds with MAO-B antagonizing activity exert a potential activity in the progression of AD.

MAOs comprise two isoenzymes—MAO-A and MAO-B—encoded on separate genes on the X-chromosome. They are characterized as flavoproteins, and both contain the redox coenzyme system flavin adenine dinucleotide (FAD) as a cofactor [7]. More than 70% similarity between the two isoenzymes was reported [8]. Additionally, numerous reviews have been published, rigorously emphasizing the catalytic effects of the latter oxidases [9,10]. Overall, the MAOs are responsible for the oxidation of aliphatic and aromatic amines to the corresponding aldehydes. They are found to bind to the outer membrane of mitochondria in most cell types. MAO-A deactivates serotonin, while both isoenzymes are heavily involved in the neutralization of dopamine [11]. Despite the significant similarities between the two isoforms, their active sites greatly differ from each other (discussed in Section 1.3) [12]; hence, selective inhibitors towards one of the two enzymes could be designed. Clorgyline, or clorgiline, is an irreversible and selective inhibitor of MAO-A. Typical selective MAO-B inhibitors approved for the treatment of neurodegenerative disorders are deprenyl, safinamide, and rasagiline [13]. The detailed crystallographic characteristics of the flavin-dependent enzyme are provided in a separate section in this work.

Molecular docking is a heavily applied structure-based computational technique, which is emerging as an eminent approach in the processes of hit discovery and lead optimization. In general, it examines the interactions between two molecules (most of the time, the complex is composed of a ligand and a receptor), and a prediction of the binding affinity and the poses of the ligands is made [14]. The in silico method provides data about the active conformations and the energy profiles of the complex, and it scores the occurring interactions (hydrogen interactions, Van der Waals contacts, electrostatic interactions, desolvation effects, entropy changes, etc.) [15]. Currently, molecular docking is used for lead optimization (the structural modeling of the more potent and selective derivatives of the ligand) and hit identification [16]. Additionally, docking strategies are employed in the process of in silico fragment-based ligand design (FBLD) [17]. The growing number of resolved crystal structures of MAO-B enzymes with various co-crystallized ligands, together with the rapidly evolving computing power, has opened the door for the swift and facilitated utilization of molecular docking as a virtual technique for the visualization of major interactions between novel MAO-B inhibitors and the active site of the latter enzyme [18].

In the next sections, the principles of the various docking software, essence, and limitations of the rapidly evolving virtual technique—molecular docking—as well insight into the crystal structure of MAO-B, are reviewed. As a final stage, the docking protocols of a recently synthesized and chemically diverse set of MAO-B inhibitors are reported.

### 1.1. Types of Molecular Docking

Initially, the binding connections between a receptor and a ligand were based on the proposed “key–lock” theory [19]. However, since the suggestion of this thesis by Fisher in 1894, the concept of ligand–receptor interactions has experienced significant alterations. In 1958, Koshland et al. modified the theory and introduced the idea of flexible ligands and enzymes, which was accepted as the leading principle in the formation of a ligand–receptor complex [20]. Nevertheless, the primary docking protocols employed a shape-fitting approach, which is plausible, considering the underdeveloped computational hardware at that time [21]. The rigid geometric algorithm was the first to be implemented in the docking program developed by Kuntz et al. [22]. Despite the great scientifically high value of the paper, several limitations of the rigid simulation protocols were described. The authors discussed the lack of comprehensively, highly resolved receptors, the general assumption of geometrically fitting ligand and active cavity, and finally the absence of any flexibility during the simulations. Moreover, the basic scoring functions, such as surface area burial, solvation free energy, and electrostatic interaction energy, were reported to have a low success rate for the predictions of the binding affinities. The development of flexible docking protocols has been declared as a suitable solution for these problems; however, the exponential increase in the computational efforts hindered the utilization of flexible simulations [23]. Since 1995, reports applying flexible docking have been growing exponentially, mainly considering the unremitting advancements in hardware power [24,25]. Monte Carlo, in-site combinatorial search, ligand buildup, and site mapping with fragment assembly are the current approaches for a flexible ligand docking protocol. A suitable article comparing the differences between rigid and flexible ligand–receptor docking algorithms has been published by Sheng-You Huang [26], and readers are kindly forwarded to this paper.

Despite the extensive application of molecular docking simulations in CADD; it has several challenges. Firstly, the flexibility of the receptor has been one of the most arduous tasks to solve. Simulations with fully flexible receptors are still impracticable, since a significant growth in the number of degrees of freedom is obtained, which drastically increases the computational expenses [27]. Recent suitable advances in that area have been made by the introduction of ensemble docking [28] and the implementation of side chain flexibility [29]. However, the application of the latter techniques does not always lead to enhanced enrichments and reliable results [30]. Another major problem is the approximative character of the calculative function of the scoring algorithms, which leads to false-positive results [31,32]. A suitable solution is the application of thermodynamic methods for the estimation of the binding affinity of ligands; however, these simulations are time consuming and hardware dependent [33]. Moreover, calculations with MM/GBSA and MM/PBSA contain several approximations. such as the calculations of water molecules and the lack of conformational entropy [34,35]. Finally, the presence of water molecules during the molecular docking simulations should also be examined [36,37,38].

### 1.2. Docking Software

Over the last 25 years, numerous docking programs have been developed and applied in the virtual screening processes. The search for minimum ligand–receptor energy serves as a foundation in the establishment of docking software. Each docking program is constructed out of one or several searching and scoring algorithms. The search algorithm places the ligands in the active site of the receptor in the search for active conformations. They are broadly classified into systematic (incremental construction and conformational search), stochastic (Monte Carlo, genetic algorithms, and tabu search), and simulation algorithms (molecular dynamics and energy minimization) [39]. After generating a series of poses in the active site of the receptor, the docking program scores all the solutions, considering the occurred interactions. The utilization of a proper scoring function is a major factor for the success of the simulation, since the latter evaluates the poses of the ligand in the receptor and provides corresponding fitness scores which are then ranked. Four major types of scoring algorithms have been defined—force field, empirical, knowledge-based, and, most recently, machine-learning-based [40,41]. A comparative assessment of eleven scoring functions, which are part of a diverse set of docking software, has been discussed by Renxiao Wang et al. [42]. The paper unambiguously concluded that by no means are the current scoring functions perfect; herein, further work in that field needs to be conducted.

A recent study by Zhe Wang et al. [43] compared the sampling and the scoring abilities of ten docking programs (LigandFit, Glide, GOLD, MOE Dock, Surflex-Dock, AutoDock, AutoDock Vina, LeDock, rDock, and UCSF DOCK). GOLD and LeDock displayed the best results in the detection of correct ligand poses. GOLD acquired an accuracy of 59.8%, while the percentage of correct ligand conformations obtained from LeDock was calculated to be 80.8% [43]. The study noted the significant robustness of Glide (XP) and GOLD. Some of the most used (based on citations) docking programs, with their basic characteristics, are provided in Table 1. A common approach in the docking algorithms of the software include a trade-off between accuracy and hardware resources; therefore, almost all programs treat the ligand as flexible and the receptor as rigid. The exception is the docking program GOLD, which has an option to treat the receptor as flexible [39]. Considering the importance of active water molecules during docking simulations [36,37], all of the discussed software shown in Table 1 have the option to include active waters during the calculations.

### 1.3. Structure and Active Site of MAO-B

**Figure 1:**
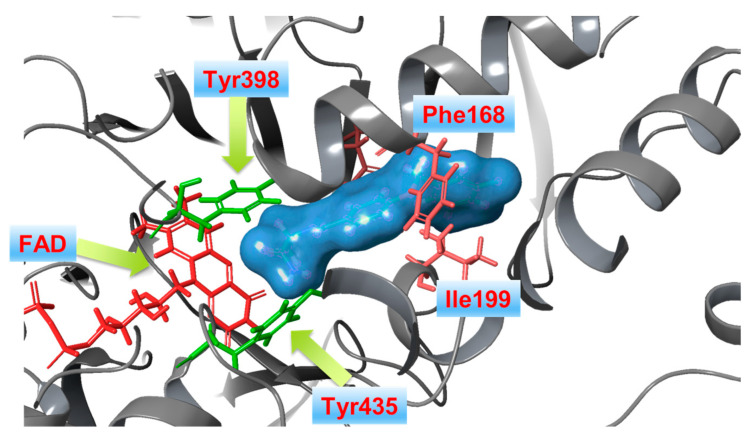
X-ray structure of *h*MAO-B in complex with safinamide (blue mesh) (PDB: **2V5Z**). The co-enzyme flavin-adenine dinucleotide (FAD) is depicted in red. The amino acids Tyr398 and Tyr435, forming the “aromatic cage”, are shown in green.

Numerous papers have reported the binding site of MAO-B [50,51]. The analyses postulated the appearance of three functional domains identified as entrance cavity, substrate pocket, and “aromatic cage” (Figure 1) [52]. All, except the “aromatic cage”, have been described as hydrophobic, and they participate in the forming of the active site cavity, which has a volume of approximately 700 Å^3^ [53]. Moreover, the volume of the entrance gauge has been computed to be 290 Å^3^, whereas the substrate-binding cavity has been reported to be approximately 490 Å^3^. Both pockets are separated by the formation of a “loop” constructed out of four amino residues: Phe168, Leu171, Ile199, and Tyr326. The position of the former sequence is essential for the formation of a stable complex, as it assists the ligand to reach the active substrate cavity [54]. Furthermore, it has been discussed that Ile199 acts as a “gate”, and the alterations of open/close state are driven by the presence of a ligand. In general, it has been hypothesized that when a ligand is absent, Ile199 is in the closed conformation, and it physically blocks the space between the two cavities [55]. Moreover, stabilizing interactions between MAO-B inhibitors and Ile199 through Van der Waals bonds have been reported [56,57]. A study by Hubalek et al. [58] has discussed the role of Ile199 towards the MAO-B selectivity. FAD together with Tyr398 and Tyr435 design the “aromatic cage” of the enzyme [59]. Li et al. [60] have discussed the plausible effect of the “aromatic cage” on amino groups comprising ligands. The authors have noted possible polarization of the amine lone pair and, thus, increased nucleophilicity which promotes activated ligand. Further study of this catalytic effect has confirmed the former theory [61].

In addition, the interaction of active MAO-B inhibitors with Tyr326 residue is reported to be essential for the selectivity [62]. As a part of the described “loop”, Tyr326 is located near the junction of the entrance and substrate gorges. The phenyl moiety of the former amino acid acts as a wall in the substrate cavity which adds additional steric constrain in the active site of MAO-B [58].

A recent crystallographic study has emphasized a potential inhibition of the imidazoline site of MAO-B [63]. The presence of tranylcypromine in the substrate cavity has modified the Ile199 position into “closed” and has allowed firm and selective inhibition of MAO-B by 2-(2-benzofuranyl)-2-imidazoline (2-BFI). The notable growth of ligand affinity when the imidazoline binding site has been focused had provided the foundation for further experiments in that area [64].

**Figure 2:**
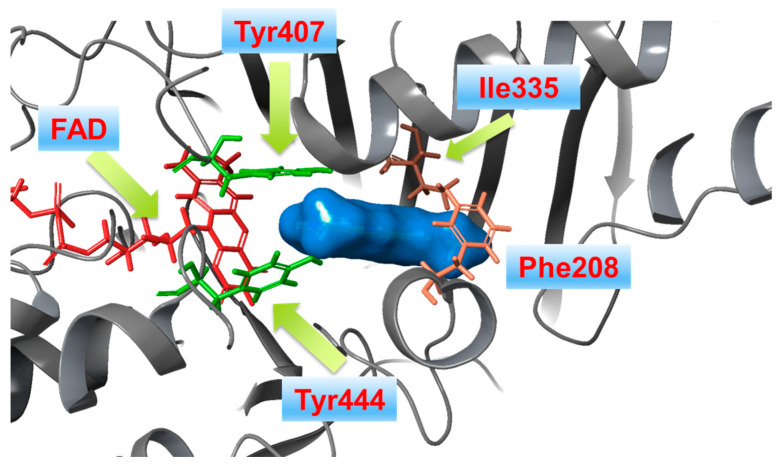
X-ray structure of *h*MAO-A in complex with harmine (blue mesh) (PDB: **2Z5X**). The co-enzyme flavin adenine dinucleotide (FAD) is depicted in red.

The current interest of some research groups is the discovery of selective and reversible MAO-B inhibitors [65]. High selectivity towards the enzyme is essential due to the possible risk of a hypertensive crisis caused by the inhibition of MAO-A [66,67]. Both isoenzymes share 72% sequence identity, however *h*MAO-A’s active site consists of a single hydrophobic cavity with a size of 550 Å^3^ which is smaller compared to the active gorge of MAO-B. Additionally, a specific “loop” conformation formed with the *h*MAO-A residues 210–216, differ from that of *h*MAO-B and is used as a basis for the development of novel and selective MAO-A inhibitors [68]. The active amino acids Tyr398 and Tyr435, included in the formation of the “aromatic cage” of MAO-B, are replaced with Tyr407 and Tyr444, respectively, in the active site of MAO-A (Figure 2) [54].

The formation of similar gorge which recognizes and interacts with ligands comprising aromatic fragments explains why MAO-A has a similar affinity for aromatic substrates. A major determinants for high inhibitor selectivity against MAO-A are the active amino residues Phe208 and Ile335 [69].

The importance of reversible MAO inhibitors in the treatment of neurodegenerative disorders has been investigated and discussed by Park et al. [70]. Moreover, the design of new MAO-B inhibitors still exists as a major problem, considering the ineffectiveness of the current therapy schemes. A proper illustration for that is a controlled trial conducted in 1997 which revealed disappointing results after a long-term Alzheimer’s treatment with the selective and reversible MAO-B blocker selegiline [71].

The main purpose of this paper was to review all recent advances in molecular docking protocols of novel and prominent MAO-B inhibitors. The compounds were classified by their chemical nature, where in the corresponding tables we specified all the structures used in this study, together with the utilized docking software and the corresponding PDB codes for each reviewed case.

## 2. Docking Studies of Recently Synthesized MAO-B Inhibitors

### 2.1. Chalcones

**Figure 3:**
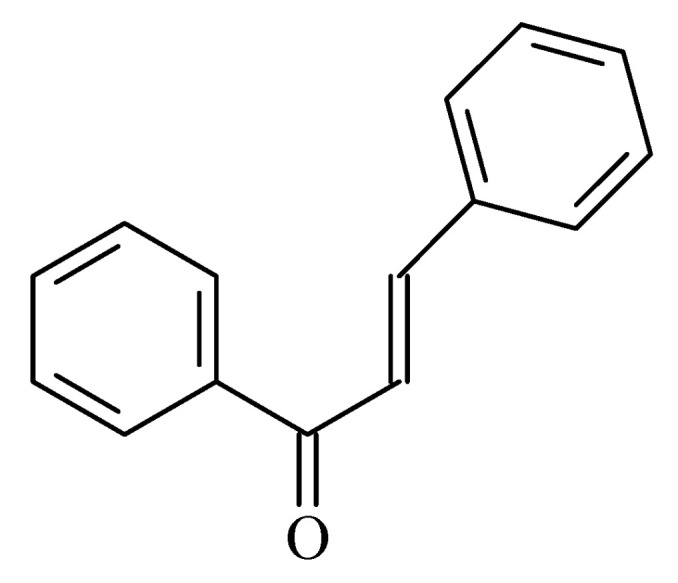
Chemical structure of chalcone.

Chalcone-based compounds (Figure 3) have various therapeutic applications considering the diverse pharmacological targets they could affect [72]. Recently, the design and synthesis of MAO-B inhibitors containing a chalcone scaffold have rapidly been growing [73]. A major scientific effort in that area has been achieved by Bijo Mathew, confirmed by his involvement in numerous published papers concerning the MAO-B activity of chalcone derivatives. Various PDB structures have been applied in the reported docking protocols. Mathew et al. discussed the synthesis and molecular docking of eleven chlorinated thienyl chalcones (Table 2, item 1) containing MAO-B and MAO-A inhibitory effects [74].

**Figure 4:**
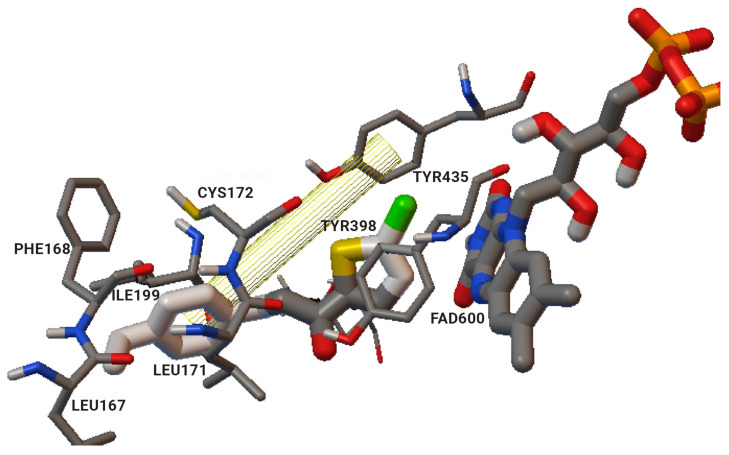
Interaction of chlorinated thienyl chalcone ligand with the active site of *h*MAO-B (PDB: **2BYB**). The yellow mesh indicates π-π stacking interaction [74].

The authors used AutoDock4.2 as software for the molecular simulation and X-ray structure of *h*MAO-B in complex with deprenyl (PDB: **2BYB**) obtained from the Protein Data Bank. Water molecules were deleted and the covalent bond between the co-crystallized ligand and N5-atom of FAD in the structure of **2BYB** was corrected. The center of the grid was set at N5 atom of FAD. The thiophene moiety was placed in the substrate cavity, while the aromatic ring of the chalcones interacted with Tyr435 through Van der Waals bonds (Figure 4).

**Figure 5:**
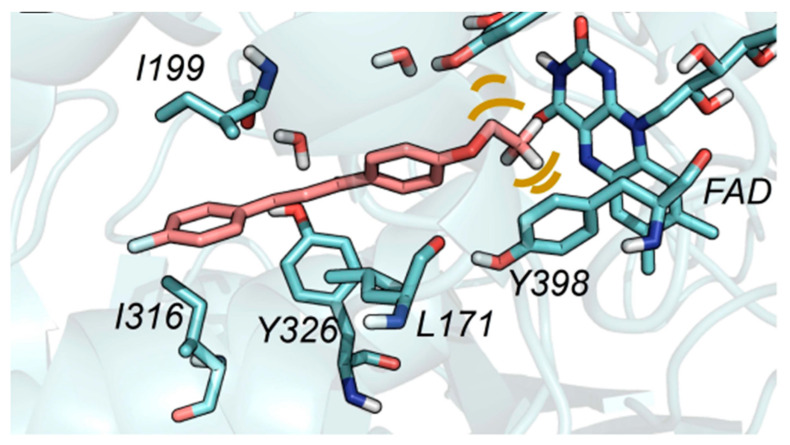
The active amino residues of MAO-B are presented as an cyan blue, and the ethoxylated chalcone is depicted in red [76].

Further stabilization via π-π interactions between the ligands and the aromatic cage was noted. It was concluded that the ethyl substitution in the phenyl ring led to higher MAO-B affinity. The same group published a paper of the synthesis and computational studies of brominated thienyl chalcones (Table 2, item 2) [75]. The utilized docking protocol of this study corresponds to the one described in the previous case. Among ethyl, methyl, methoxy, and dimethylamine groups placed at the para-position of the brominated thienyl chalcones, the dimethylamino moiety showed the best potency and selectivity. Furthermore, the docking software predicted that the indicated groups were projected into the entrance cavity. Further examination showed the dimethylamino moiety, as the bulkiest group, spanned both the entrance and the substrate pockets. π-π interactions between the aromatic ring of the chalcone and the “aromatic cage” were also considered. Glide docking, for the evaluation of the MAO inhibitory activity of eleven ethoxy substituted chalcones (Table 2, item 3), was applied by Lakshminarayanan et al. [76]. The ligands were first synthesized, and the virtual screening was carried out on an X-ray MAO-B structure (PDB: **2V5Z**). Hydrophobic interactions between the ligands and the amino acids Ile199, Ile316, and leu171 were examined (Figure 5).

Compound 1-(4-ethoxyphenyl)-3-(4-fluorophenyl)prop-2-en-1-one displayed the best MAO-B inhibitory potential. The acquired docking poses show that the methyl group of the p-ethoxy moiety was placed in the aromatic cage of the MAO-B structure. When subjected to virtual screening in the active site of MAO-A, the interaction with FAD was absent due to a steric hindrance.

**Figure 6:**
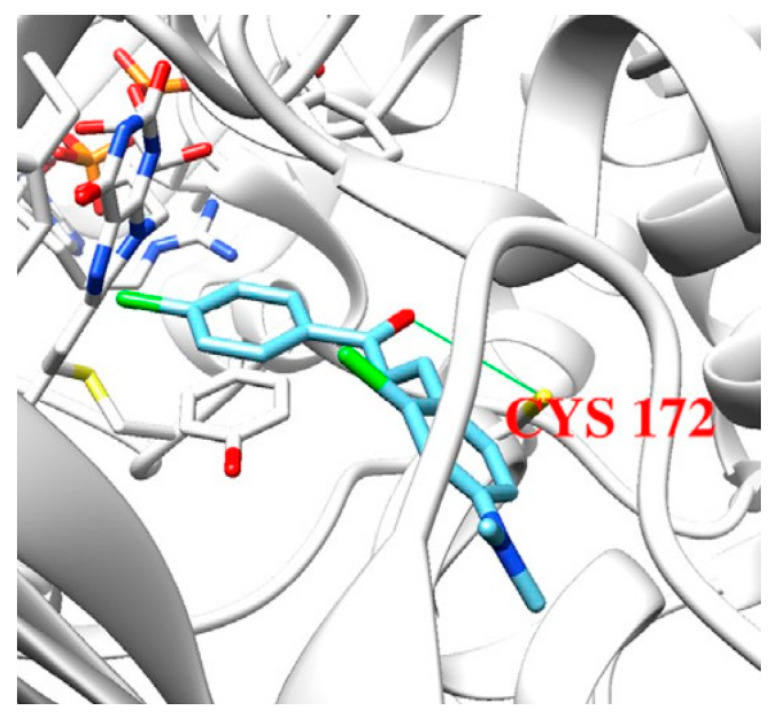
Docking simulations of 2,4’-dichloro-4-dimethylaminochalcone in MAO-B [77].

John Oh et al. [77] studied the MAO-B inhibition effect of various natural and synthetic chalcones (Table 2, item 4). The molecular docking simulation was carried out by AutoDock Vina using the 3D structure of MAO-B with co-crystallized pioglitazone (PDB: **4A79**). Further docking studies towards MAO-A and AChE receptors were performed, and it was discussed that the analyzed chalcones have higher selectivity towards MAO-B. The dimethoxy chalcone oxime with fluoro substitution in the acetophenone moiety showed the most prominent selectivity and blocking activity towards MAO-B. It was observed that the high MAO-B activity was due to the hydrophobic π-π interactions between Tyr326 and the ligand. Furthermore, the formation of a hydrogen bond between the carbonyl oxygen of chalcones and Cys172 of MAO-B increased the binding affinity of the ligand (Figure 6).

**Figure 7:**
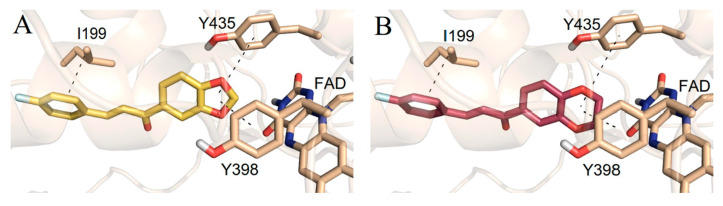
Docking poses of the top-ranked oxygenated chalcones in MAO-B. The ligands and the active amino residues are given as sticks [78]. Panel (**A**,**B**) represent the active conformations of 1,3-dioxolane and 1,4-dioxane chalcone-based molecules, respectively.

The design, synthesis, and biological evaluation of oxygenated chalcones (Table 2, item 5) were reported by Parambi et al. [78]. The crystal structure of MAO-B was downloaded from PDB: **2V5Z**. The Schrödinger suite was used for protein preparation, and eight molecules of water were preserved in the active site of MAO-B. The QM polarized ligand docking from Schrödinger Suite was applied as docking software. The docking study of the most active and selective oxygenated chalcone demonstrated that the benzoxazole group was close to the FAD and moderate–strong π-π stacking interactions with Ile199, Tyr398, and Tyr435 formed (Figure 7). An additional hydrogen bond was established between Tyr326 and the hydrogen bond acceptor of the ligand, which further confirms the important role of Tyr326 for the MAO selectivity.

### 2.2. Coumarins

**Figure 8:**
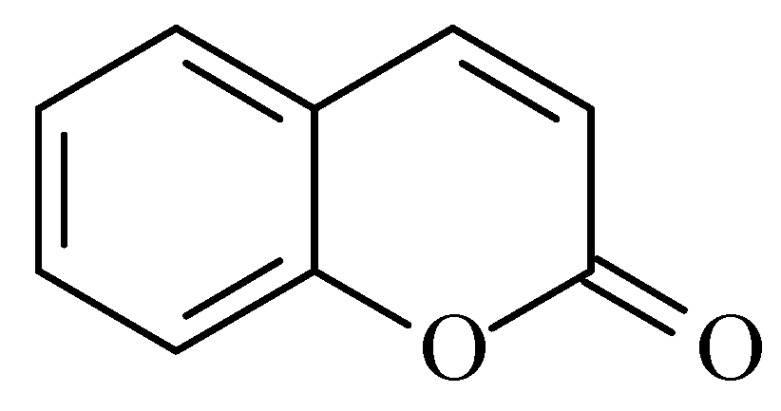
Chemical structure of coumarin.

Coumarins are structural isomers of chromones and were investigated for MAO, cholinesterase, and aromatase antagonizing activities (Figure 8). The latter multitarget properties were utilized for the treatment of neurodegenerative disorders [79]. Recent papers have demonstrated the selectivity and potency of coumarins as MAO-B inhibitors [80].

**Figure 9:**
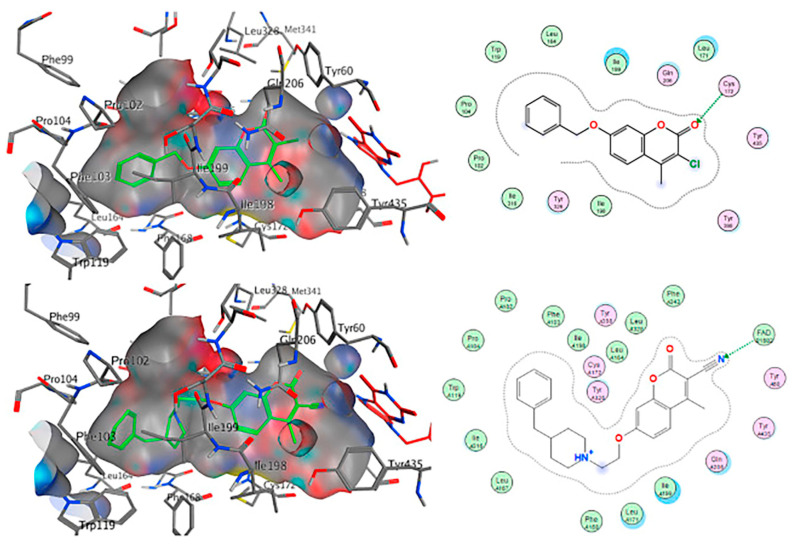
The active conformations of coumarin derivatives in the active site of MAO-B. FAD is depicted in red and the compounds are shown in green [81].

Joubert et al. reported the synthesis and virtual screening results of 7-substituted coumarin derivatives (Table 3, item 1) [81]. Molecular operating environment (MOE) software was applied for the docking study, and *h*MAO-B crystal structure with co-crystallized 7-(3-chlorobenzyloxy)-4-(methylamino)methylcoumarin (PDB: **2V61**) was used. The obtained data show that the top solutions situated their coumarin moiety in the polar region of the substrate cavity (Figure 9).

Moreover, a hydrogen bond between the carbonyl group of most compounds and Cys172 was observed. Van der Waals interactions between benzyl and the N-benzylpiperidine side chain of the molecules and the hydrophobic entrance cavity further stabilized the complex.

Another study reported the synthesis and determination of the MAO-B inhibitory activity of coumarin–dithiocarbamate hybrid compounds [82]. The research group applied Molecular Operating Environment (MOE, version 2008.10) software for the molecular modeling. The study concluded that the coumarin moiety (Table 3, item 2) was in the substrate cavity and further stabilized by binding to Tyr60, Phe343, Tyr398, and Tyr435. The dithiocarbamate part of the molecule was situated in the entrance cavity. The most active compound of the coumarin–dithiocarbamate explored class was with an IC_50_ value of 0.101 µM. Eight compounds isolated from glycyrrhiza uralens, also known as Chinese licorice, were subjected to a molecular study and examined for their monoamine oxidase and cholinesterase inhibitory activities [83]. It was found that liquiritigenin has a moderate MAO-B inhibitory constant (IC_50_ = 0.098 µM). The virtual screening was carried out with AutoDock Vina, and MAO-B with co-crystallized pioglitazone (PDB: **4A79**) was downloaded from PDB. The active site grid was set as defined by the binding site of co-crystallized pioglitazone. The work demonstrated that in the MAO-B active site, no hydrogen bonds were formed. However, a promising correlation between in vitro and in-silico data was obtained.

Repsold et al. analyzed the MAO-B and AChE inhibitory properties of new lead compounds with coumarin moiety (Table 3, item 3) [84]. Piperidine, morpholine, thiophene, and erucic acid were used for the conjugation with coumarin. The software utilized in that research was Accelrys^®^ Discovery Studio^®^ V3.1.1, and an X-ray of MAO-B with the co-crystallized selective inhibitor (7-(3-chlorobenzyloxy)-4-(methylamino) methyl coumarin) was taken from PDB. The virtual screening indicated a reasonable MAO-B blocking activity of the coumarin–morpholine ether. It demonstrated a crucial p-interaction with Tyr398 and hydrogen bond with Cys172.

**Figure 10:**
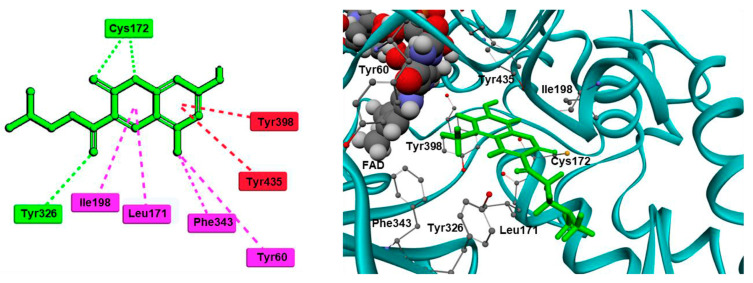
Active conformation of a coumarin Mannich base in the active site of MAO-B. Conventional hydrogen bonds are shown as green, dashed lines; π-π stacking interactions as red, dashed lines; π-alkyl interactions as purple, dashed lines [86].

In a recent study, our research group [85] validated a docking protocol of novel coumarins utilizing GOLD 5.3 as the docking software. We used the PDB: **1S3B**. The most potent ligands formed stable complexes after the formation of a hydrogen bond with FAD600. Several hydrophobic interactions with amino residues Leu171, Cys172, Ile199, and Tyr326 were also observed. Discovery Studio 2016 was utilized in the molecular docking of novel Mannich bases of 3-acetyl-7-hydroxyl coumarins (Table 3, item 4) by Tao et al. [86]. After the initial in vitro determination of MAO-B activity, two compounds showed promising antagonism towards the enzyme and were further evaluated. The coumarin moiety was orientated in the substrate cavity between Tyr398 and Tyr435. Moreover, the complex was further supported by a hydrogen bond between Cys172 and the oxygen atom of the lactone ring (Figure 10).

**Table 3:** Structures of the reported coumarins evaluated as MAO-B inhibitors utilizing various docking software and PDB codes.

S. No.	Structure	MAO-B Inhibitor Activity	Docking Program	PDB Code	Ref.
1.	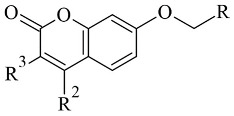	IC_50_ 0.0005—28.5 (μM)	Molecular Operating Environment (MOE)	2V61	[81]
2.	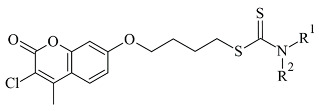	IC_50_ 0.101—47.16 (μM)	Molecular Operating Environment (MOE)	2V61	[82]
3.	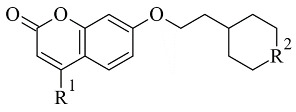	IC_50_ 0.372—94.32 (μM)	Discovery Studio (DC)	2V61	[84]
4.	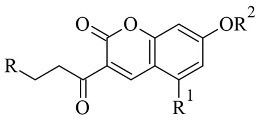	IC_50_ 4.62—>200 (μM)	Discovery Studio (DC)	4A79	[86]
5.	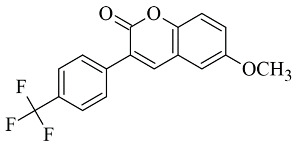	IC_50_ 56—8476 (nM)	Glide	2V60	[87]

**Figure 11:**
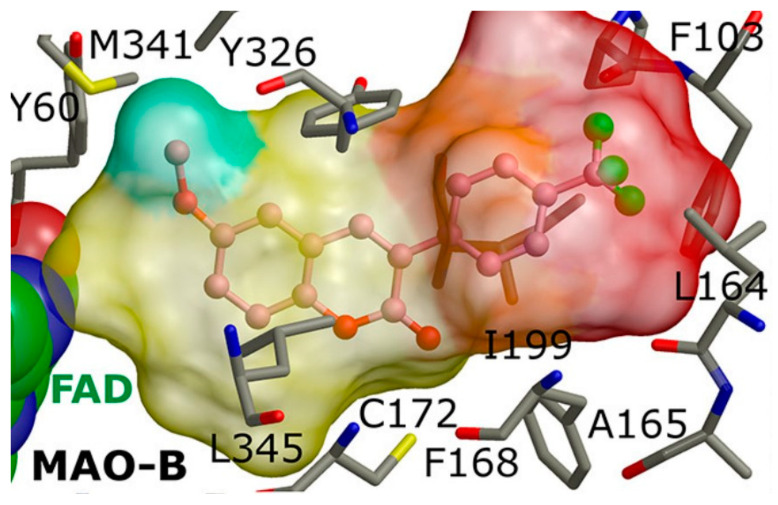
The active site of MAO-B with the docked 3-phenylcoumarin derivative [87].

Rauhamaki et al. designed a virtual library of 3-phenylcoumarin derivatives (Table 3, item 5) and subjected them to a virtual screening using Glide [87]. The most promising compounds were synthesized and assessed in vivo for MAO-B activity. From the computational study, the importance of the 3-phenyl moiety of the coumarins for the MAO-B inhibitory activity was analyzed. The hydrophobic interaction of the coumarin and 3-phenyl ring with the substrate pocket was established as an essential part of the MAO-B inhibitory activity (Figure 11).

Interestingly, the 3-phenylcoumarins were reversed in the active site compared to the validated co-crystallized ligands. Moreover, the authors discussed an atypical hydrogen bond of the 2C-carbonyl group with the thiol moiety of Cys172; however, the latter interaction did not inflict any alterations.

### 2.3. Chromones

**Figure 12:**
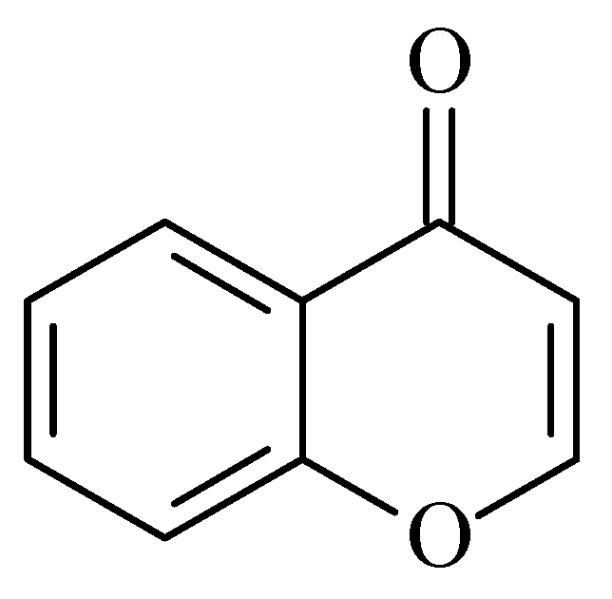
Chemical structure of chromone.

Recently published works have demonstrated the prominent chromone scaffold (Figure 12) for the design of novel MAO-B inhibitors [88]. The majority of the chromone derivatives demonstrated high selectivity towards MAO-B, which has been explained with the smaller active gauge in MAO-A [89]. An emphasis was placed on the chromone-3-phenylcarboxamide moiety [90].

**Figure 13:**
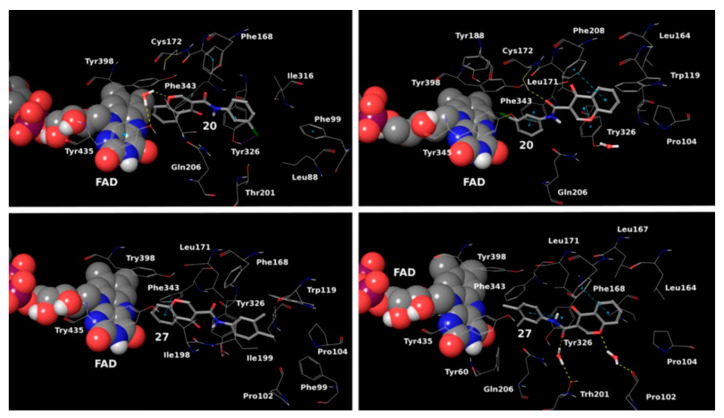
MD trajectory frames of active chromone-3-phenylcarboxamide inhibitor in the active site of *h*MAO-B [91]. Ligands are given in polytube while the FAD cofactor is depicted in the spacefill.

Reis et al. reported the synthesis and the molecular docking of a small library of chromone-3-phenylcarboxamides (Table 4, item 1) with MAO-B blocking activity [91]. Glide was utilized as docking software for the virtual screening of the potent chalcones. Interactions of the chalcone derivatives with Phe102 and Tyr435 have been noted (Figure 13).

Additionally, the water-bridged bond of the carbonyl oxygen with Tyr201 was observed. The paper examined the steric hindrance when the bulky structure was set into the active site of MAO-B. The latter ligands lacked strong interactions and, therefore, were assessed as weaker inhibitors. The work further discussed the solvent effect after MD simulations and trajectories inspections. Moderate correlation with the experimental data was observed. Another paper published by Reis et al. discussed the synthesis and the screening of selective MAO-B chromone derivatives (Table 4, item 2) [92]. The most promising compounds were virtually evaluated using the Glide module in Schrödinger Suite 2016-3. The grid was centered at N5 of FAD. As presumed, the phenylcarboxamide moiety was in the aromatic cage. The larger ligand pocket in the active site of MAO-B is accountable for facilitated ligand fitting. MAO-A has a smaller active cavity which leads to a steric hindrance and therefore inability in the formation of stable interactions with the ligands.

Genetic Optimization of Ligand Docking (GOLD) 3.0.1 was applied for the virtual screening of novel 7-benzyloxy-8-carboxamide-chromone derivatives (Table 4, item 3) with selective MAO-B inhibitory activity [93]. The authors noted that the occurrence of numeral hydrogen bonds between the functional groups of the chromone derivatives and the amino residues from the active site drastically increased the potency of the ligands. The hydrogen bonds were explained with the presence of several groups (mainly oxygen and nitrogen atoms) propagating these types of interactions. The molecular docking revealed the optimal amide aryl and benzyl orientations, in the active site, for a prominent MAO-B activity.

Virtual screening of newly synthesized chromone-donepezil hybrid compounds (Table 4, item 4) as carried out by Wang et al. [94]. The research group applied MOE 2008.10 as a docking program for the evaluation of the binding modes. The results displayed π-π interactions between the benzyloxy group of the ligands and the aromatic cage strictly with Tyr398 and Tyr435. An additional hydrogen bond formed between Tyr326 and the amide carbonyl of one of the compounds. Moreover, the quaternary nitrogen of the piperidine ring was involved in hydrogen bond interaction with Glu84.

**Figure 14:**
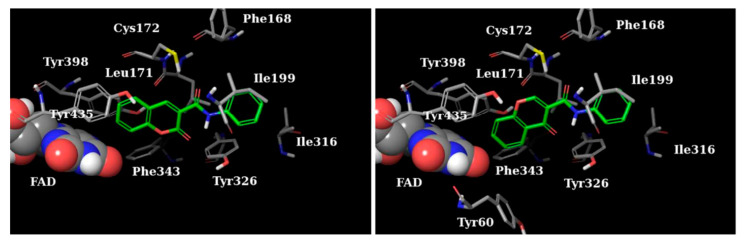
Active conformations of 3-aminomethylidene-2,4-chromandiones into the active site of *h*MAO-B [95].

A docking study of chromone derivatives (Table 4, item 5) was carried out using Discovery Studio 3.1 [95]. X-ray structure of MAO-B was downloaded from a protein data bank (PDB: **2Z5X**). It is noted that the most prominent molecules were 3-aminomethylidene-2,4-chromandiones. Two isomers of the most potent chromone with MAO-B blocking activity have been analyzed. As expected, π-π interaction of the chromone with Tyr398 and Tyr435 was observed (Figure 14).

**Figure 15:**
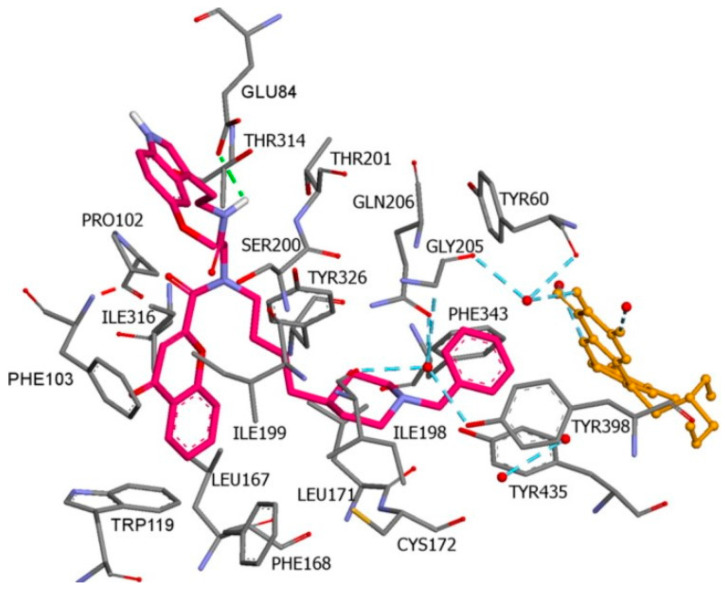
A Donepezil + chromone + melatonin hybrid derivative in the active site of MAO-B. The active amino acids are color coded [96].

Furthermore, a notable hydrogen bond between the carbonyl group located at C2 of the chromone ring and Ile199 was examined. The molecular docking concluded that both cis and trans isomers display MAO-B inhibitory activity. Donepezil + chromone + melatonin hybrids (Table 4, item 6) were synthesized and virtually evaluated for their implementation in Alzheimer’s disease [96]. X-ray MAO-B with co-crystallized safinamide was used as a model target (PDB: **2V5Z**). AutoDockVina was applied as docking software. π-π interactions were observed between the phenyl ring of the hybrid derivatives and Tyr60, Gly205, Phe343, and Tyr398 (Figure 15).

A hydrogen bond formed between the amide group and Glu84. The indole ring was situated at the beginning of the hydrophobic entrance pocket. The chromone moiety was positioned in the entrance cavity as well.

### 2.4. Indanones

**Figure 16:**
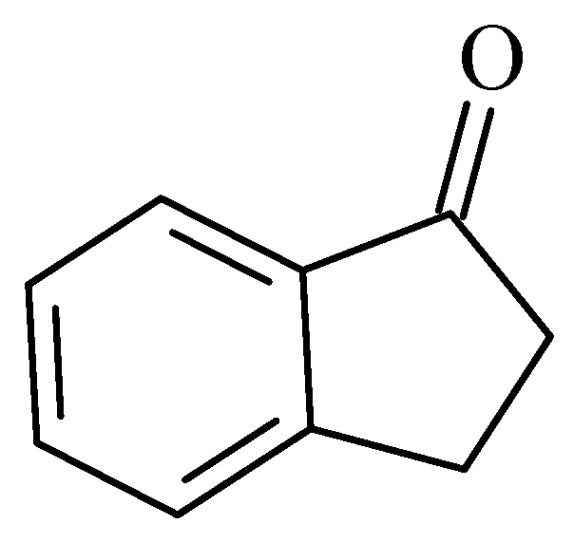
Chemical structure of 1-indanone.

A recently published review described the vast pharmacological activities of Indanone derivatives (Figure 16) including antiviral, antimicrobial, and anticancer properties [97]. In addition, some indanones have shown significant MAO-B inhibitory capacity which proposes a prominent anti-Alzheimer activity [98]. Nel et al. evaluated 2-benzylidene-1-indanone derivatives (Table 5, item 1) for MAO-B inhibitory activity [99].

**Figure 17:**
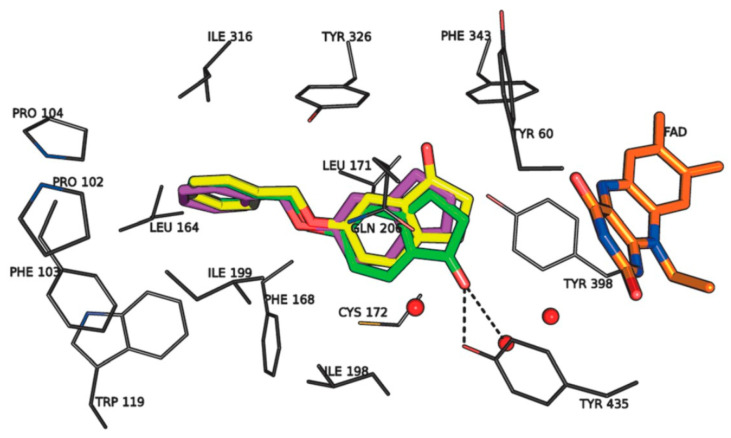
The top-ranked docking solutions of 1-indanone derivatives in the active site of MAO-B [100].

The authors have used the **2V5Z** crystal structure of MAO-B and Discovery Studio 3.1 (Accelrys) have been utilized for the virtual screening. The docking studies have shown that the indanone moiety was situated in the substrate cavity, while the benzylidene groups were facing the entrance cavity. π-π interactions with Tyr398 and Tyr435 have been described and further VdW binding stabilized the structures in the hydrophobic entrance pocket. In 2015, Mostert et al. [100] evaluated the MAO effect of numerous 1-indanone derivatives (Table 5, item 2). For the virtual screening **2V5Z** PDB file and Discovery studio version, 3.1 were used. It was found that the best solutions had their 1-indanone moiety bind in the substrate cavity of MAO-B (Figure 17).

The Van der Waals forces were described as the main interaction between the active site of the enzyme and the ligands. Moreover, hydrogen bonding was detected between the 5-benzyloxy-1-indanone (**2a**) and water molecules. The authors have suggested that the formation of the latest hydrogen bond is due to the position of the carbonyl group towards the entrance cavity.

2-Heteroarylidene-1-indanone derivatives (Table 5, item 3) with MAO activity have been synthesized and subjected to molecular docking [101]. 1-indanones could be considered as cyclic analogs of heterocyclic chalcones and similar MAO inhibitor functions have been reported. The docking studies have been carried out with the CDOCKER docking function of Discovery Studio 3.1 (Accelrys) in the active site of MAO-B (PDB: **2V5Z**). The validation of the method was done by redocking of the co-crystallized ligand. From the study, it was found that the indanone moiety was situated in the aromatic cage, while the heteroaryl ring was facing the entrance cavity. The authors noted that π-π and Van der Waals interactions were dominant. They examined 2-Heteroarylidene-1-indanones are characterized as reversible MAO inhibitors.

### 2.5. Pyrazoles

**Figure 18:**
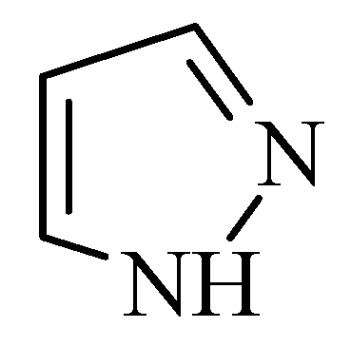
Chemical structure of pyrazole.

**Figure 19:**
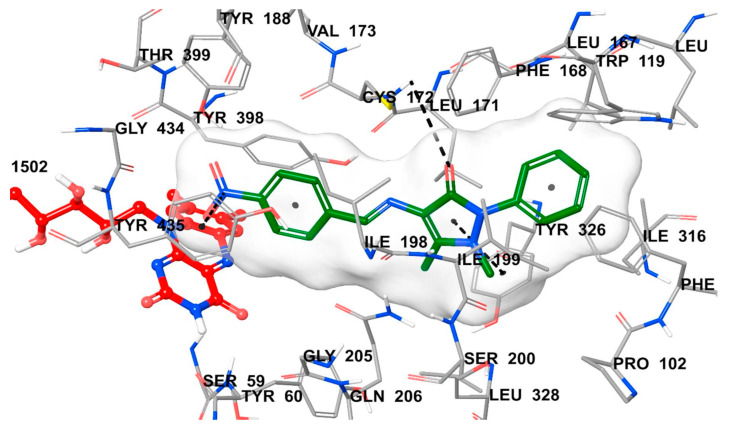
The interacting mode of 1,3,5-trisubstituted pyrazoline in the active gorge of MAO-B [104]. The inhibitor and the active residues are presented by tube model.

Pyrazole-based compounds (Figure 18) are prominent dual MAO-B and AChE inhibitors. The MAO inhibitor activities of 1,3,5-trisubstituted pyrazolines have been evaluated and studies have shown a decent effect on the inhibition of the enzyme [102]. Moreover, derivatives with unsubstituted N1 pyrazoles have been researched for selective MAO-B activity and they have been determined as potent inhibitors [103]. Recent docking protocols are discussed below. Tok et al. have utilized molecular docking protocols for the most active novel pyrazole derivative (Table 6, item 1) in the active sites of MAO-B and AChE [104]. The structure was situated close in the vicinity of FAD (Figure 19).

**Figure 20:**
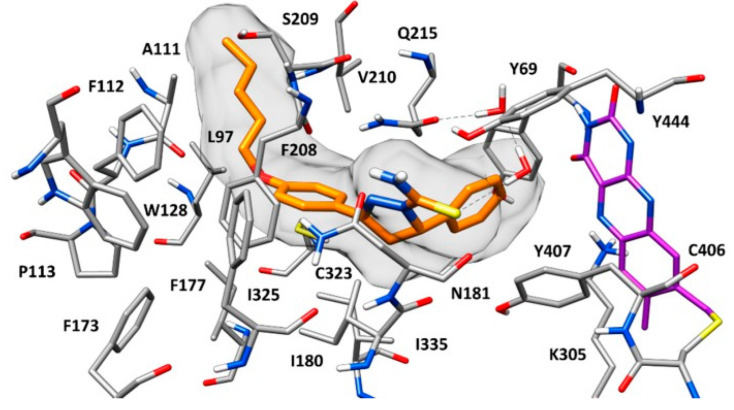
Active conformation of (S) enantiomer of 1-thiocarbamoyl-3,5-diphenyl-4,5-dihydro-(1H)-pyrazole in the active site of MAO-B [105]. The flavin group of FDA is shown in purple, and the molecular surface of the ligand is shown in gray.

Furthermore, the virtual observation determined the presence of a hydrogen bond between the carbonyl group of the compound and the amino residue Cys172. It is noted that the cation-π interaction between electron-withdrawing groups and Tyr435 further increases the activity of the complex. π-π interaction between Tyr326 and the phenyl moiety was also detected. AutoDock 4.2 docking was applied for virtual screening of a small library of 4,5-dihydropyrazoles with thioamide moiety (Table 6, item 2) [105]. *h*MAO-B (PDB: **4A79**) was downloaded from PDB. Both (S) and (R) stereoisomers were subjected to docking protocols. In both forms, the phenyl ring attached at 5C in the pyrazole core was situated between Tyr398 and Tyr435 and π-π bonding occurred (Figure 20).

The central phenyl ring interacted with Leu171, Cys172, Ile199, and Tyr362 through hydrophobic bonds. The (S)-enantiomer indicated lower MAO-B inhibitory activity because of the missing hydrogen bonds between the ligand and FAD. However, the terminal phenyl group forms strong π-π bonds with Tyr398 and Tyr435 is compensative for some of the dropped activity. The work emphasized the requirement of an NH_2_ group as an H-donor and a sulfur atom as H-acceptor for higher MAO-B activity. Further elevation of MAO-B affinity was observed when a long, hydrophobic chain was present. The work concluded significant higher selectivity of the ligands towards MAO-B.

Grewal et al. synthesized and docked novel pyrazole carboxamide derivatives (Table 6, item 3) with dual inhibitor activity for MAO-B and acetylcholinesterase [106]. The interaction was evaluated in the active site of MAO-B (PDB: **2V61**). The binding affinities of the ligands were compared to the co-crystallized ligand. AutoDock Vina was applied for the computational screening. The molecules with lower δG were selected and further assessed in vivo for MAO-B activity. Further investigation of the binding affinities and poses was performed with PyMOL. A good correlation between in silico and in vivo screening was obtained. 3,5-Disubstituted 2-pyrazoline derivatives (Table 6, item 4), with a phenyl moiety, were synthesized, and a molecular docking protocol was applied towards the crystal structure of MAO-A [107]. The docking software Glide and the X-ray structure of MAO-A (PDB: **2Z5X**) were used for the docking study. The paper provided worthy information concerning the structural requirements for the selective inhibition of MAO-A.

### 2.6. Xanthines

**Figure 21:**
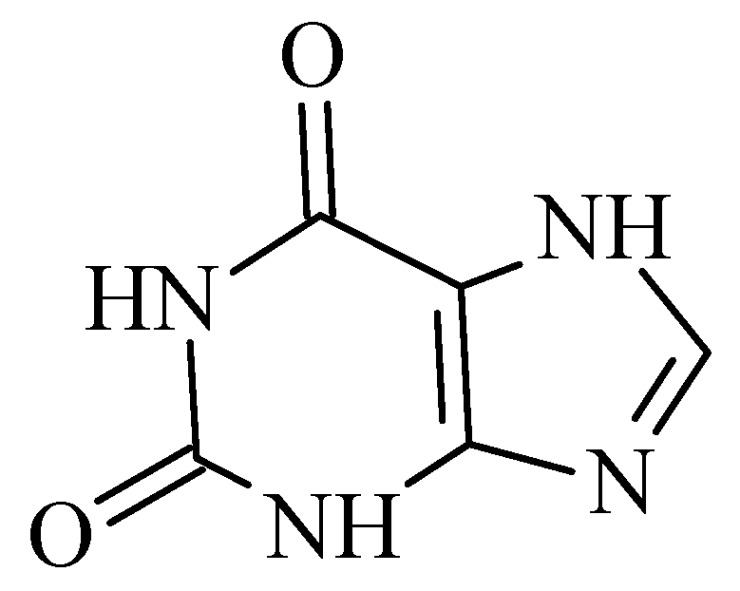
Chemical structure of xanthine.

**Figure 22:**
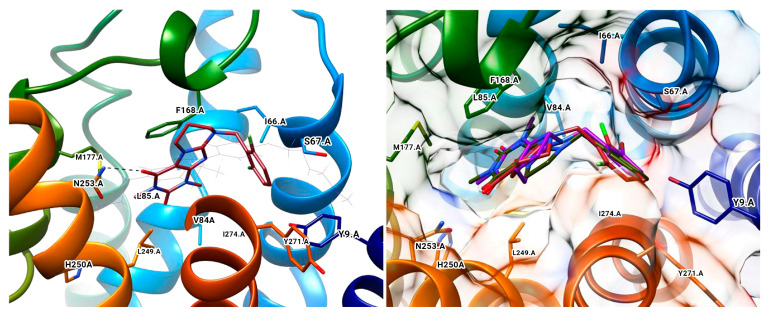
Calculated active conformations of xanthine-based compounds in the active gorge of MAO-B [112].

Derivatives of the xanthine (Figure 21) have been extensively studied for their application in the treatment of neurodegenerative disorders [108]. The big majority of these molecules display both A2 and MAO-B inhibitory properties [109]. The reversible and potent MAO-B activity of caffeine derivatives was recently stated [110]. A major drawback in the docking protocols of xanthines derivatives is the absence of X-ray MAO-B structures with co-crystallized xanthine derivatives. However, several docking reports of novel xanthine derivatives with MAO-B inhibitory function have recently been published and cited below. All the docking protocols utilized the X-ray structure of MAO-B with co-crystallized safinamide considering some similarities to the purinedione core [111]. A series of N9-benzyl substituted xanthines (Table 7, item 1) were synthesized and evaluated in vitro and in silico [112]. Glide was applied as docking software and the crystal structure of MAO-B was obtained from PDB: **2V5Z**. The rankings of the ligands were determined by their position and the binding affinity with the amino acids in the binding pocket of MAO-B. The work reported π-π interactions of a purinedione moiety with Tyr398 and Tyr435 (Figure 22).

Two different binding modes have been detected. In one of which the substituted phenyl group has been pointing towards the entrance cavity and the second one which has shown that the ligand was in close vicinity to FAD. In the latter case the purinedione core is flipped horizontally and which additionally stabilizes the complex by the formation of π-π stacking intecations with Tyr 398. Moreover, the OH-group of Tyr188 forms a hydrogen bond with the carbonyl group of the ligand. In some poses, halogen bonds with Tyr201 have been noted. Furthermore, it was noted that the toxicity of the derivatives increased with the structural modifications in the aromatic ring.

Koch et al. [113] designed 1,3-substituted xanthines (Table 7, item 2) and the subsequent docking showed promising adenosine- and monoamine-oxidase-blocking abilities. Schrodinger Suites was used for the ligand and protein preparation. Glide was utilized for the virtual docking. The crystal structure of MAO-B with a co-crystallized safinamide was downloaded from PDB. The grid box of the protocol followed the coordinates of the safinamide in the active site of MAO-B. The final binding energy of the derivatives was recorded and examined. It was noted that Tyr326 played a major role in the interaction with the ligands. Similar bonds have been found when safinamide binds to MAO-B [114]. The authors noted that the “head-to-tail” orientation could occur because of the close energetic levels between the two poses.

**Table 7:** Structures of the reported xanthine derivatives evaluated as MAO-B inhibitors utilizing various docking software and PDB codes.

S. No.	Structure	MAO-B Inhibitor Activity	Docking Program	PDB Code	Ref.
1.	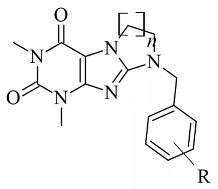	IC_50_ 0.083—111 (μM)	Glide	2V5Z	[112]
2.	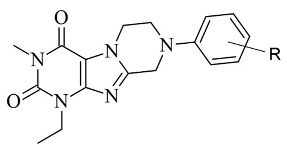	IC_50_ 106—136 (nM)	Glide	2V5Z	[113]
3.	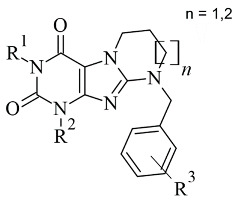	IC_50_ 83—1241 (nM)	Glide	2V5Z	[115]

**Figure 23:**
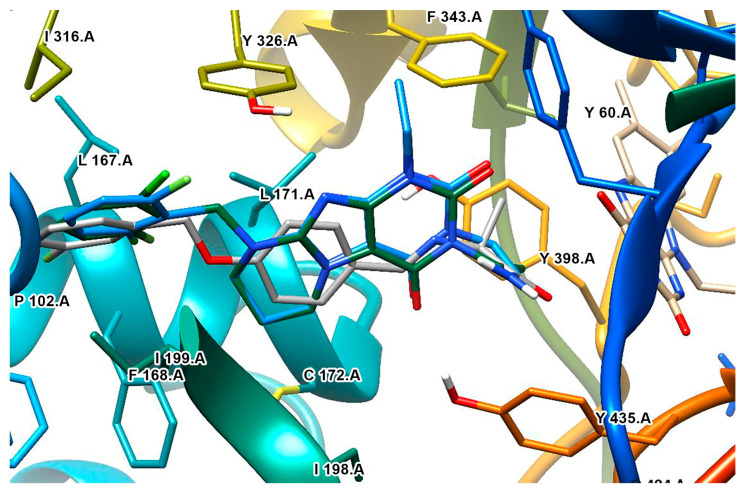
Active superimposed conformations of xanthine derivatives in MAO-B [115].

Schrödinger Maestro Suite was utilized for the molecular docking of novel annulated xanthine derivatives (Table 7, item 3) [115]. The procedure was validated with the redocking of co-crystallized safinamide to the crystal structure of MAO-B (PDB: **2V5Z**). As foreseen, the purinedione moiety was in the aromatic cage between Tyr398 and Tyr435 and the phenyl group was facing the entry cavity (Figure 23).

An additional hydrogen bond was noted between Tyr435 and the carbonyl oxygen placed at N6. When subjected to screening towards MAO-A X-ray structure, the ligands did not show any activity which demonstrated high selectivity towards MAO-B.

### 2.7. Miscellaneous Compounds

In 2016, Li et al. discussed the design, synthesis, and evaluation of novel donepezil-like compounds for the treatment of Alzheimer’s disease [116]. A molecule with a pyridine moiety (Table 8, item 1) was described as the most potent MAO-B, MAO-A, AChE, and BuChE inhibitor. The computational screening was completed with Molecular Operating Environment (MOE) version 2008.10 (Chemical Computing Group, Montreal, Canada). The X-ray structure of MAO-B with a co-crystallized ligand has been downloaded (PDB: **2V60**). The N-benzylpiperidine moiety was in the substrate cavity where π-π stacking between the benzene ring and Tyr398 was examined. In vitro study determined the inhibitory value (Table 8, item 2) as 3.14 µM.

**Figure 24:**
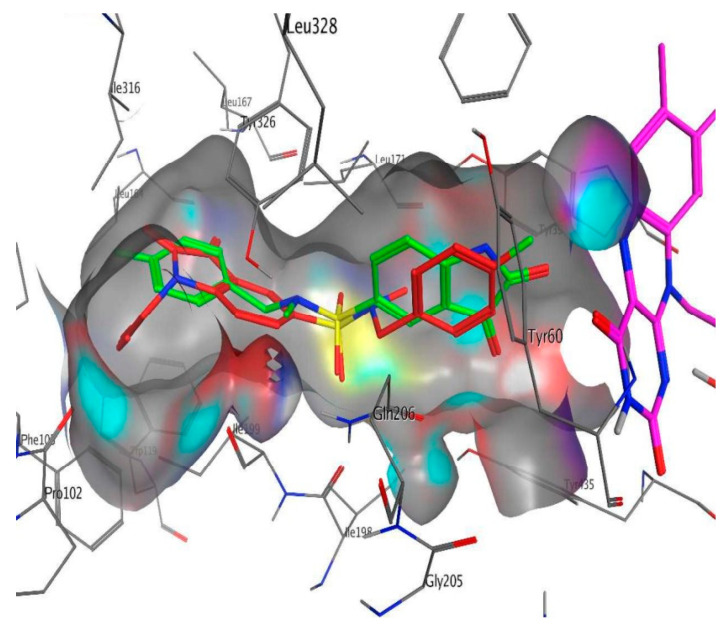
Binding modes of two sulfonyl isatin derivatives in the active gorge of MAO-B [113].

The docking module of Molecular Operating Environment (MOE), Version 2015.10. software as applied for docking studies of novel sulfonyl isatin derivatives (Table 8, item 2) [117]. The MAO-B X-ray structure was downloaded from PDB (code: **2V5Z**). It was noted that the isatin was situated in the “aromatic cage” of the active site, and the fluorophenyl sulfonyl side chain faced the entrance cavity (Figure 24).

The Ile199 was in the open conformation. Additional Van der Waals bonds in the hydrophobic entrance pocket stabilized the complex. A drastic reduction in the MAO-B activity was observed when isatin was in the entrance cavity. 

Tripathi and Ayyannan discussed the docking protocol of novel 2-amino-6-nitrobenzothiazole hydrazone derivatives (Table 8, item 3) [118]. AutoDock 4.2 and *h*MAO-B with code **2V5Z** were utilized for the docking study. The 6-nitrobenothiazole moiety in the most active compounds was situated in the entrance cavity, while the hydrazone groups were located between the entrance and the substrate gouges. The complex was further stabilized by hydrogen bonds and π-π interactions. The in vitro and in silico experiments showed good correlation.

**Figure 25:**
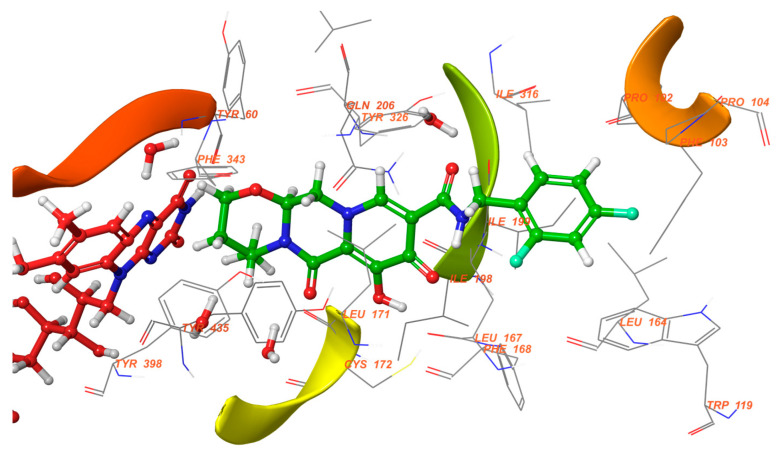
Major intermolecular interactions between the active site of **2V5Z** and dolutegravir [119]. The active ligand is shown in green, and the active amino acids are depicted in orange.

A study conducted by our research group discovered novel MAO-B inhibitors by in silico drug repurposing [119]. After in vitro evaluations, we noted that dolutegravir posed moderate MAO-B blocking capacity at 1 µM concentration. The docking simulations were carried out using two docking programs, GOLD 5.3 and Glide, in the active site of **2V5Z**. The benzyl moiety of dolutegravir was facing the entrance cavity, while the core structure was located in the “aromatic cage” of MAO-B (Figure 25).

Tyr398 and Tyr435 amino residues formed π-π interactions with the 1,3-oxazinane group, while FAD established a carbon hydrogen bond with the same fragment. Several π–alkyl interactions were formed between the 2,4-difluorobenzyl moiety and the active amino acids Leu164, Ile199, and Ile316.

## 3. Conclusions

In this review, we addressed the major interactions between the active site of MAO-B and numerous novel inhibitors against the former enzyme. In all the discussed cases, the significant role of the “aromatic cage”, for the selectivity and reversibility of the ligands, was evident. The importance of several active amino residues in the formation of stable H-bonds was also noted. All cited papers specified the docking programs and the PDB codes of the corresponding X-ray structures. In some works, moderate correlations between in silico and in vitro data were observed, which provides high reliability for the obtained poses. Self- and cross-docking was frequently applied to assess the accuracy of the docking protocols.

## Figures and Tables

**Table 1:** Commonly utilized docking software.

Program	Company/Designer	License Terms	Search Algorithm	Score Function
AutoDock [44]	D. S. Good sell and A. J. Olson The Scripps Research Institute	Free for Academic use	Genetic algorithm Lamarckian genetic algorithm	Auto Dock (Force-field)
AutoDock Vina [45]	Dr. Oleg Trott Molecular Graphics Lab (CCSB), The Scripps Research Institute	Free for Academic use	Gradient optimization algorithm	United-atom scoring function
DOCK [46]	I. Kuntz University of California, San Francisco	Free for Academic use	Shape fitting; AMBER/GBSA and AMBER/PBSA for Dock 6.0	Chem Score, GB/SA solvation Scoring
FlexX [47]	T. Lengauer and M. Rarey Bio Solve IT	Commercial Free evaluation	Incremental Build	FlexXScore, PLP, Screen Score, Drug Score
Glide [48]	Schrödinger Inc.	Commercial	Monte Carlo Sampling	Glide Score, Glide Comp
GOLD [49]	Cambridge Crystallographic Data Centre	Commercial	Genetic Algorithm	ChemPLP, GoldScore, ASP, Chemscore

**Table 2:** Structures of the reported chalcones evaluated as MAO-B inhibitors utilizing various docking software and PDB codes.

S. No.	Structure	MAO-B Inhibitor Activity	Docking Program	PDB Code	Ref.
1.	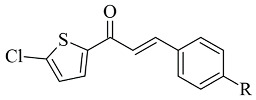	*Ki* 5.52—0.49 (μM)	AutoDock 4.2	2BYB	[74]
2.	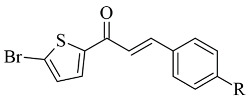	*Ki* 0.11—9.30 (μM)	AutoDock 4.2	2BYB	[75]
3.	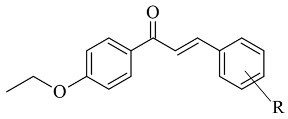	IC_50_ 0.68—28.50 (μM)	Glide	2V5Z	[76]
4	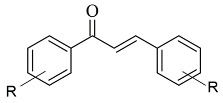	IC_50_ 0.0029—13.2 (μM)	AutoDock Vina	4A79	[77]
5.	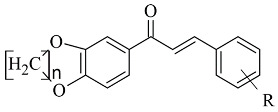	IC_50_ 0.00021—0.068 (μM)	Glide	2V5Z	[78]

**Table 4:** Structures of the reported chromone derivatives evaluated as MAO-B inhibitors utilizing various docking software and PDB codes.

S. No.	Structure	MAO-B Inhibitor Activity	Docking Program	PDB Code	Ref.
1.	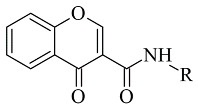	IC_50_ 1.03—7396 (nM)	Glide	2V5Z	[91]
2.	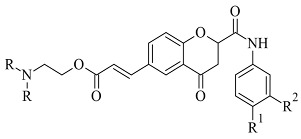	IC_50_ 0.63—3.81 (μM)	Glide	2V5Z	[92]
3.	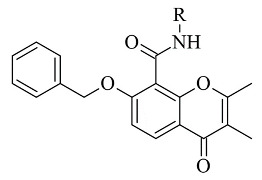	IC_50_ 0.078—2.22 (μM)	GOLD	1GOS	[93]
4.	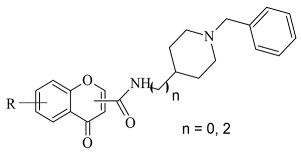	IC_50_ 0.035—52.45 (μM)	Molecular Operating Environment (MOE)	2V60	[94]
5.	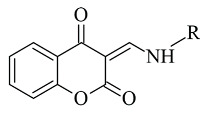	IC_50_ 5.07—621 (nM)	Discovery Studio (DS)	2Z5X	[95]
6.	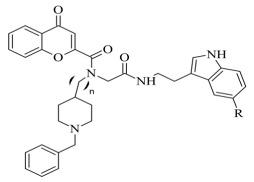	IC_50_ 15.17—80.59 (μM)	AutoDock Vina	2V5Z	[96]

**Table 5:** Structures of the reported indanone derivatives evaluated as MAO-B inhibitors utilizing various docking software and PDB codes.

S. No.	Structure	MAO-B Inhibitor Activity	Docking Program	PDB Code	Ref.
1.	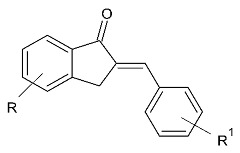	IC_50_ 0.0052—2.74 (μM)	Discovery Studio (DS)	2V5Z	[99]
2.	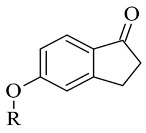	IC_50_ 0.015—0.298 (μM)	Discovery Studio (DS)	2V5Z	[100]
3.	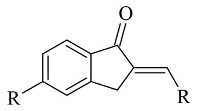	IC_50_ 0.0044—1.53 (μM)	Glide	2V5Z	[101]

**Table 6:** Structures of the reported pyrazole derivatives evaluated as MAO-B inhibitors utilizing various docking software and PDB codes.

S. No.	Structure	MAO-B Inhibitor Activity	Docking Program	PDB Code	Ref.
1.	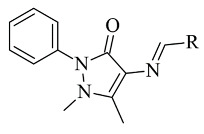	IC_50_ 0.049—0.114 (μM)	Glide	2V5Z	[104]
2.	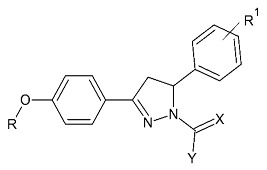	IC_50_ 0.38—>100 (μM)	AutoDock 4.2	4A79	[105]
3.	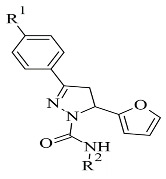	-	AutoDock Vina	2V61	[106]
4.	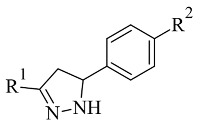	-	Glide	2V5Z	[107]

**Table 8:** Structures of some reported miscellaneous compounds evaluated as MAO-B inhibitors utilizing various docking software and PDB codes.

S. No.	Structure	MAO-B Inhibitor Activity	Docking Program	PDB Code	Ref.
1.	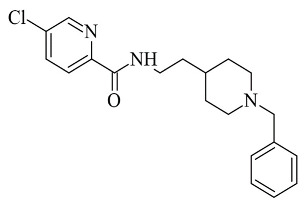	IC_50_ 2.53—94.1 (μM)	Molecular Operating Environment (MOE)	2V60	[116]
2.	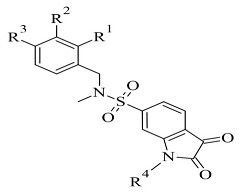	*Ki* 2.57—55.18 (μM)	Molecular Operating Environment (MOE)	2V5Z	[117]
3.	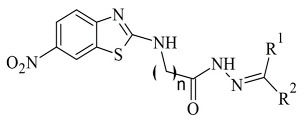	IC_50_ 0.00018—137 (μM)	AutoDock 4.2	2V5Z	[118]
